# A novel infant microbiome formula (SIM03) improved eczema severity and quality of life in preschool children

**DOI:** 10.1038/s41598-024-53848-w

**Published:** 2024-02-07

**Authors:** Oi Man Chan, Wenye Xu, Nam Sze Cheng, Agnes Sze Yin Leung, Jessica Yuet Ling Ching, Brian Leong Yuen Fong, Pui Kuan Cheong, Lin Zhang, Francis Ka Leung Chan, Siew Chien Ng, Ting Fan Leung

**Affiliations:** 1 Department of Paediatrics, The Chinese University of Hong Kong, Prince of Wales Hospital, Shatin, Hong Kong SAR China; 2 Department of Medicine and Therapeutics, The Chinese University of Hong Kong, Shatin, Hong Kong SAR China; 3 Microbiota I-Center (MagIC), Shatin, Hong Kong SAR China; 4 Hong Kong Hub of Paediatric Excellence, The Chinese University of Hong Kong, Shatin, Hong Kong SAR China

**Keywords:** Gut microbiome, Atopic dermatitis, Children, Probiotic, Bifidobacterium, Skin diseases, Paediatric research, Clinical trial design

## Abstract

Altered gut microbiome composition has been reported in children with eczema and interventions that restore beneficial bacteria in the gut may improve eczema. This open-label pilot study aimed to investigate the efficacy of a novel infant microbiome formula (SIM03) in young children with eczema. Pre-school Chinese children aged 1–5 years old with eczema received SIM03 twice daily for three months. The novelty of SIM03 consists of both the use of a patented microencapsulation technology to protect the viability of unique *Bifidobacterium bifidum* and *Bifidobacterium breve* strains identified through big data analysis of large metagenomic datasets of young Chinese children. Paired stool samples at baseline and following SIM03 were analyzed by metagenomics sequencing. Generalized estimating equation was used to analyze changes in eczema severity, skin biophysical parameters, quality of life and stool microbiome. Twenty children aged 3.0 ± 1.6 years (10 with severe eczema) were recruited. Treatment compliance was ≥ 98%. SCORing Atopic Dermatitis score decreased significantly at two months (*P* = 0.008) and three months (*P* < 0.001), while quality of life improved significantly at 1, 2, and 3 months. The relative abundance of *B. breve* and microbial pathways on acetate and acetyl-CoA synthesis were enriched in stool samples at one month (*P* = 0.0014). Children who demonstrated increased *B. bifidum* after SIM03 showed improvement in sleep loss (*P* = 0.045). Relative abundance of *B. breve* correlated inversely with eczema extent (*P* = 0.023) and intensity (*P* = 0.019) only among patients with increased *B. breve* at Month 3. No serious adverse event was observed. In conclusion, SIM03 is well tolerated. This patented microbiome formula improves disease severity and quality of life in young eczematous children by enhancing the delivery of *B. bifidum* and *B. breve* in the gut. SIM03 is a potential treatment option for childhood eczema.

Eczema is the most common chronic skin disease in infants and young children. It is an inflammatory skin condition characterised by recurrent eczematous lesions and intense itch. The Global Burden of Disease study reported that eczema affected up to 20% of children worldwide^1^. Our territory-wide survey found that close to one-third of preschool children in Hong Kong had eczema ever^2^. Eczema pathogenesis involved a complex interplay between defective epidermal barrier and microbial imbalance that led to allergen penetration that stimulated type 2 helper T cell response^3^. Young children with eczema commonly underwent the “atopic march” to asthma and allergic rhinitis by school-age. Microbiome alteration has been shown to contribute to the predisposition for and worsening of eczema^4^. Gut microbial diversity has been shown to be reduced in children with eczema compared with healthy children. Furthermore, gut microbial diversity was reported to be lower at one month postnatal among children who developed eczema at 6 and 12 months of age^5^. These children also showed reduced fecal Bifidobacterium and Lactobacillus and increased *Clostridium difficile*, *Escherichia coli* and *Staphylococcus aureus* than healthy controls^6–10^. The abundance of bifidobacteria in the gut was also found to be inversely correlated with eczema severity in children^8^.

The concept of microbiome-driven approaches to restore dysbiosis has revolutionised treatment for eczema over the past decade^11^. Nonetheless, published studies rarely analyzed any microbiota change in subjects’ stool following probiotic treatment. The gut delivery of probiotic bacteria remained unknown. Based on big data analysis of metagenomic datasets between eczematous children and healthy infants of our large-scale birth cohorts (Smart Baby and 100 K mother-baby Greater Bay cohort)^12,13^, our group invented a unique infant microbiome formula, SIM03 which is currently under patent application, for treating childhood eczema. The novelty of this invention consists of both the unique formulation of naturally occurring strains of *Bifidobacterium breve* and *Bifidobacterium bifidum* found to be deficient in our eczematous children and the adoption of a patented microencapsulation technology to protect these bacteria and enhance their gut delivery. The strains and proportions of these two bifidobacteria will be disclosed by patent office after the patent has been granted. We hypothesized that dysbiosis in eczematous children could be modulated by SIM03 via restoration of beneficial gut microbiota leading to improvement in their eczema symptoms. In an open-label pilot study, we aimed to evaluate the effectiveness and safety of SIM03 on clinical outcomes including eczema severity, quality of life and gut microbiome composition in young Chinese children with eczema.

## Subjects and methods

### Subjects

Twenty children aged 1–5 years with eczema were recruited from pediatric clinics of our university-affiliated teaching hospital in Hong Kong. Eczema was diagnosed according to the Hanifin and Rakja criteria^14^. Subjects with the following conditions were excluded: (a) received systemic immunosuppressants within six months; (b) systemic antibiotic or probiotic supplements within one month; (c) consumption of probiotic supplements and/or probiotic-containing formula milk; and (d) clinically significant chronic skin diseases. Joint Chinese University of Hong Kong-New Territories East Cluster Clinical Research Ethics Committee approved this study (reference 2022.102-T), and subjects’ parents signed informed written consent. This study was conducted in strict compliance with Good Clinical Practice according to guidelines of the International Conference on Harmonization. This study was registered as NCT05607511 in *ClinicalTrials.gov*.

### Intervention

The study product SIM03 (GenieBiome, Hong Kong) was produced under Good Manufacturing Practice and contains a mixture of naturally occurring strains of *Bifidobacterium breve* and *Bifidobacterium bifidum* (10^9^ CFU in each sachet). SIM03 used a patented microencapsulation technology to protect the viability of these bacteria in the formula. Subjects consumed one sachet of SIM03 (with milk or 10–20 ml lukewarm water) twice daily for three months. Treatment adherence was assessed at each visit by parents and also based on study diary and counting the quantity of unused product sachets.

### Clinical assessments

All children were evaluated at baseline and clinical demographics including weight and standing height were documented. Parents were given a study diary to record eczema and gastrointestinal symptoms and intake of study product. Subjects were then reviewed for study outcomes and treatment adherence at one, two and three months after starting SIM03. Eczema outcomes included (a) disease severity by SCORing Atopic Dermatitis (SCORAD) that measured both objective and subjective features of eczema^15^; (b) disease-specific quality of life (QoL) in the preceding week by Children’s Dermatology Life Quality Index (CDLQI) for subjects aged 4–5 years^16^ and Infants’ Dermatitis Quality of Life Index (IDQOL) for those aged 1–3 years^17^; and (c) skin biophysical parameters including skin hydration (SH) and transepidermal water loss (TEWL) by MoistureMeterSC and VapoMeter (Delfin Technologies, Kuopio, Finland) respectively according to our published methods^18^. Parents/carers also recorded daily bowel movements (time, frequency, and stool appearance) and stool consistency using Bristol Stool Scale (BSS)^19^ and any adverse event.

### Fecal samples

Fecal samples were collected at home by all subjects at baseline, Month 1 and Month 3 using tubes containing preservative media (cat. 63,700, Norgen Biotek, Ontario Canada), which preserved and allowed safe transportation of microbial DNA and RNA at ambient temperature eliminating sample variability. The stool sample was sent to the hospital within 24 h of collection and stored at − 80 °C refrigerators until further processing.

### Metagenomic sequencing and profiling

Fecal DNA extracted by DNeasy PowerSoil Pro Kit (Qiagen, Hilden, Germany) was subjected to whole-genome shotgun sequencing according to our published method^20^. Sequencing libraries were prepared from extracted DNA using Illumina DNA Prep (M) Tagmentation Kit (Illumina, CA, USA), and sequenced with paired-end 150 bp sequencing strategy by Illumina NovaSeq 6000 System at Microbiota I-Center (MagIC) in the Hong Kong Science Park. Raw sequence reads were filtered and quality-trimmed using Trimmomatic v0.39 and decontaminated against human genome (reference hg38) by Kneaddata (V. 0.10.0, https://bitbucket.org/biobakery/kneaddata/wiki/Home). Profiling of bacterial communities was performed using MetaPhlAn3 (v3.0.14) by mapping reads to clade-specific markers^21^. The output table contained bacterial species and their relative abundance in different levels, from kingdom to species level. The resulting data were analyzed by R v4.0.3 using ggplot2 (v3.3.5), phyloseq (v1.34.0), and vegan (v2.5–7). HUMANN v3.0.1 was used to profile the abundance of microbial metabolic pathways.

### Statistical analysis

Study outcomes were analysed by independent *t*-test or Mann–Whitney U test for numerical variables and Pearson χ^2^ or Fisher exact test for categorical variables. Changes in eczema outcomes during this 3-month study were analyzed by generalized estimating equation. Deseq2 (v1.30.1) was used to normalize data into relative log expression for the functionality of stool metagenomics and to identify differential microbial functional pathways. The effects of SIM03 on microbial pathways were identified by DESeq2 and confirmed with multivariate analysis by linear models (MaAsLin). Differences in the improvement in eczema severity between subjects with and without an increase in bifidobacteria species were compared by Pearson χ^2^ or Fisher exact test. Correlations between SCORAD score and relative abundances of probiotics contained in SIM03 were determined by Spearman’s rank correlation. All statistical tests were performed two-sided using SPSS version 28.0 (IBM, Armonk, NY), and an effective *P*-value < 0.05 was considered statistically significant.

## Results

Twenty children aged 3.0 ± 1.6 years consented to participate in the study. Table 1 summarizes subjects’ demographics, personal and clinical features at baseline. The majority of subjects had moderate-to-severe eczema (SCORAD ≥ 25). About half of them were taking nutritional supplements other than probiotics. SIM03 was well tolerated and treatment adherence was 99.5 ± 0.7%.

**Table 1:** Demographic, personal and clinical features of our study participants.

Characteristic	Result*
Male	8 (40)
Age, year	3.0 ± 1.6
Delivered by vaginal route	14 (70)
Breastfeeding ever	20 (100)
Exclusive breastfeeding (≥ 90%) ever	10 (50)
Duration of breastfeeding, month	13.4 ± 9.9
Current intake of nutritional supplements	9 (45)
Paternal educational level	
Secondary	9 (45)
College and above	11 (55)
Maternal educational level	
Secondary	8 (40)
College and above	12 (60)
History of physician-diagnosed allergic diseases other than eczema	
Any allergic disease	19 (95)
Food allergy	18 (90)
Allergic rhinitis	8 (40)
Anthropometric parameters	
Body weight, kg	13.4 ± 3.2
Standing height, cm	93.7 ± 12.2
Head circumference, cm	48.5 ± 2.1
Total SCORAD score (0–103)	25.6 (12.2–53.9)
Eczema severity by SCORAD score	
Mild (< 25)	6 (30)
Moderate (25–49)	4 (20)
Severe (≥ 50)	10 (50)

*Expressed in number (percentage), median (interquartile range) or mean ± standard deviation as appropriate.

**Figure 1:**
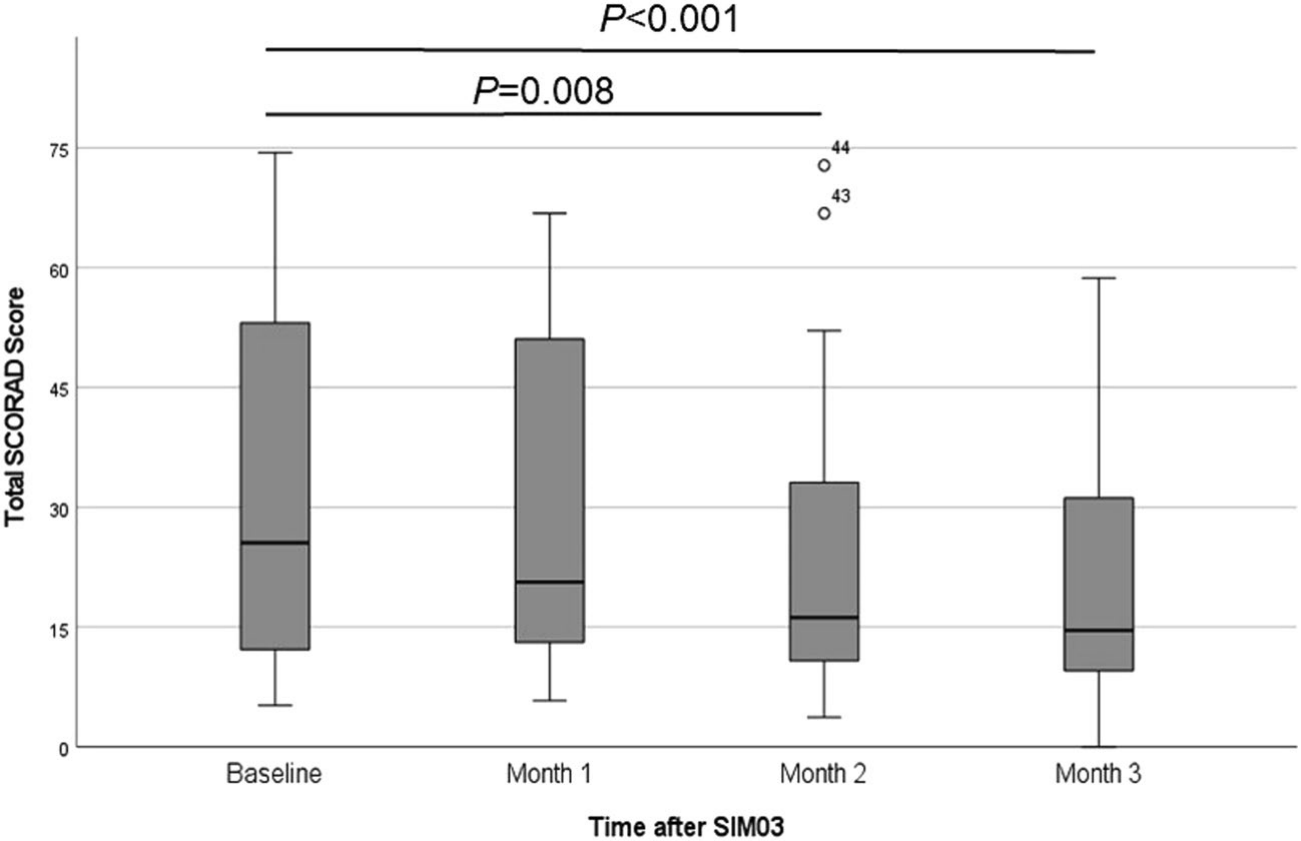
Changes in eczema severity as assessed by total SCORAD score during three months of SIM03 treatment.

The median (interquartile range) of total SCORAD score significantly decreased from 25.6 (12.2–53.9) at baseline to 16.2 (10.6–33.9) at two months (*P* = 0.008) and 14.6 (8.7–32.3) at three months (*P* < 0.001) (Fig. 1). Table 2 shows changes in different SCORAD components, QoL scores and skin biophysical parameters. Improvements in SCORAD were observed for both objective and subjective components at two months (*P* = 0.033 and 0.002) and three months (*P* = 0.003 and < 0.001). Quality of life also improved by IDQOL in children aged 1–3 years (*P* = 0.014, 0.002 and 0.013 at 1, 2 and 3 months respectively) and by CDLQI in those aged 4–5 years (*P* = 0.011, 0.011 and 0.008 for 1, 2 and 3 months respectively). Both SH and TEWL remained the same during study. Most participants (> 70%) had bowel opening at least once a day, while stool frequency and consistency were static over the 3-month period (Table S1).

**Table 2:** Changes in SCORAD components, quality of life scores and skin biophysical parameters in our subjects.

Parameter	Result*			
	Baseline	Month 1	Month 2	Month 3
Eczema severity				
Objective SCORAD (extent and intensity)	14.6 (7.5–42.9)	13.7 (8.6–40.3)	11.8 (7.4–21.9)^†^	9.0 (7.3–22.9)^§^
Extent, % BSA	8.8 (3.1–24.0)	7.0 (2.9–27.5)	5.5 (2.3–15.6)	6.5 (2.0–14.3)
Intensity	3.5 (2.0–9.8)	3.5 (2.3–9.5)	3.0 (2.0–5.0)^†^	2.0 (2.0–5.5)^§^
Subjective SCORAD (sleep loss and pruritus)	10.0 (4.5–14.0)	7.0 (5.3–10.8)	5.0 (2.0–12.0)^§^	6.0 (0.8–9.5)^¶^
Sleep loss by VAS	5.5 (2.3–7.0)	3.0 (1.3–6.8)	2.0 (0–6.5)^¶^	2.5 (0–7.0)^¶^
Pruritus by VAS	4.8 (2.3–7.0)	4.0 (1.3–6.8)	3.5 (2.0–7.3)	2.5 (0–7.0)^†^
Skin biophysical parameters				
SH, %	40.0 (21.4–51.2)	42.2 (21.7–54.3)	31.8 (24.7–46.5)	28.1 (21.5–38.6)^‡^
TEWL, g/m^2^h	10.4 (9.1–13.7)	9.9 (8.7–15.3)	11.8 (9.7–16.3)	10.4 (8.7–12.1)
Quality of life assessment				
Total IDQOL score	10.0 (4.0–15.0)	7.0 (4.0–13.0)^†^	5.0 (3.0–7.0)^§^	4.0 (3.0–13.0)^†^
Total CDLQI score	15.0 (9.5–17.5)	12.0 (6.5–17.0)^†^	11.0 (7.0–13.0)^†^	8.0 (6.5–18.0)^‡^

*BSA* body surface area, *CDLQI* Children’s Dermatology Life Quality Index, *IDQOL* Infant’s Dermatitis Quality of Life Index, *SCORAD* SCORing Atopic Dermatitis, *SH* skin hydration, *TEWL* transepidermal water loss, *VAS* visual analogue scale.

* Expressed in median (interquartile range).

^†^ *P* < 0.05; ^‡^ *P* < 0.01; ^§^ *P* < 0.005; ^¶^ *P* < 0.001 when compared with values at baseline.

Seven adverse events due to diarrhoea (loose stool for 6 times), roseola, rash over face and neck, upper respiratory tract infection and common cold were reported, but none was judged by investigators to be related to SIM03. No serious adverse event was observed.

**Figure 2:**
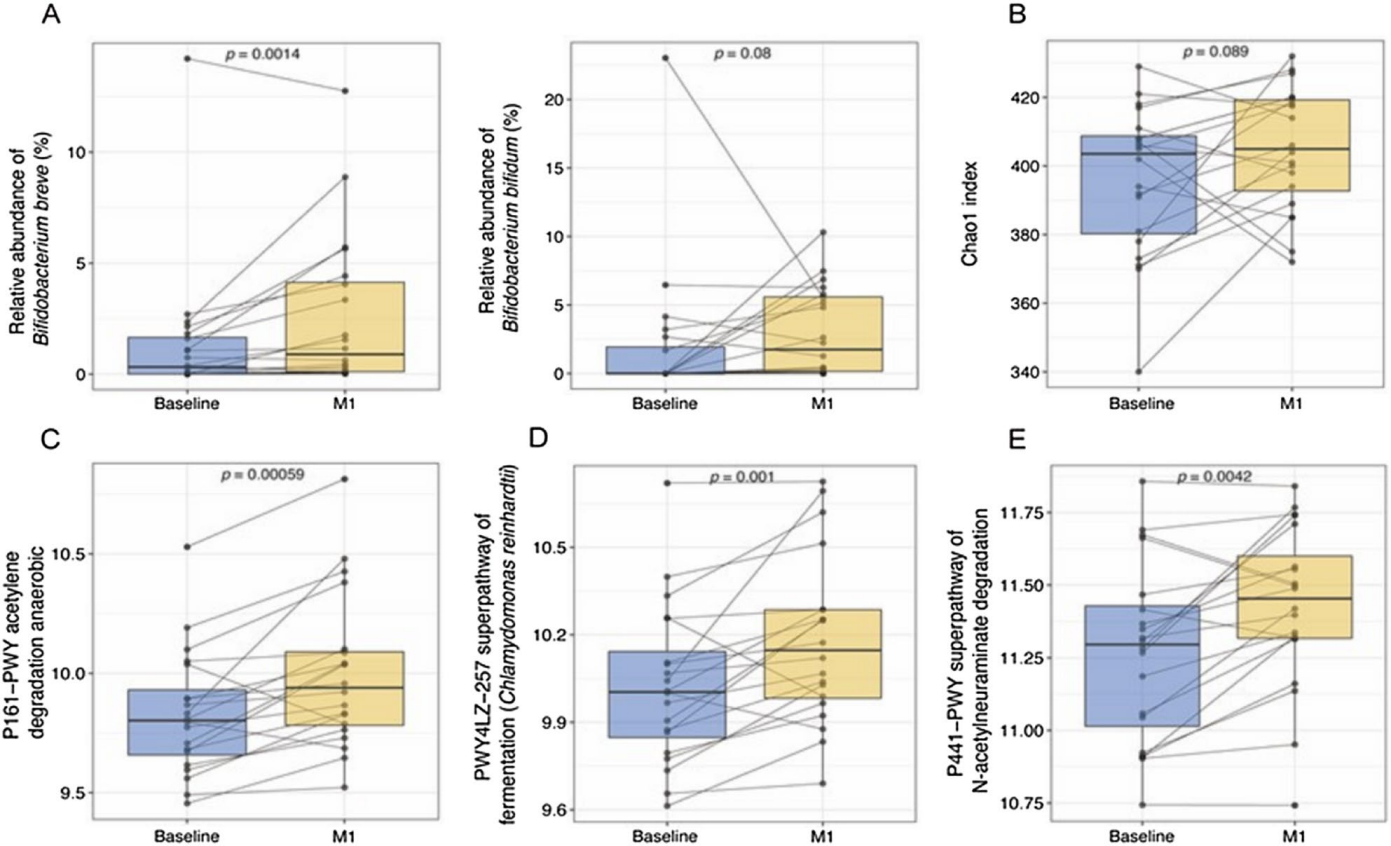
Alterations in stool microbiota and microbial pathways post SIM03 treatment. (**A**) Differences in the relative abundance of *B. breve* and *B. bifidum* between baseline and one month post-SIM03 analyzed by Wilcoxon signed-rank test. (**B**) Difference in Chao1 index of microbial pathways between baseline and one month post-SIM03. (**C**-**E**) Differences in microbial pathways between baseline and one month post-SIM03 as analyzed by both Deseq2 and MaAsLin.

Fecal samples were collected and subjected to metagenomic sequencing at baseline and one and three months after SIM03 treatment. Compared with baseline sample, there was an increase in the relative abundance of *B. breve* (*P* = 0.0014; Fig. 2A) and *B. bifidum* (*P* = 0.080; Fig. 2A) contained in SIM03 after one month of treatment. There was also a marginal increase in the richness of overall microbial pathways as indicated by Chao1 index at one month (*P* = 0.089, Fig. 2B). Three microbial pathways mainly contributed to acetate and acetyl-CoA syntheses, namely acetylene degradation, N-acetylneuraminate degradation and superpathway of fermentation (*P* < 0.05 for all) were enriched after SIM03 treatment (Fig. 2C-E) while microbial pathways related to short chain fatty acids such as butyrate remained unchanged (Table S2). Next, we examined differences in microbial pathway profiles between patients with or without increase in *B. bifidum* at Month 1 after SIM03. Four pathways significantly increased among those with increased *B. bifidum* (Table S3). Notably, the increase in superpathway of menaquinol-8 biosynthesis III (PWY-7992) remained significant after adjustment for multiple statistical testing.

**Figure 3:**
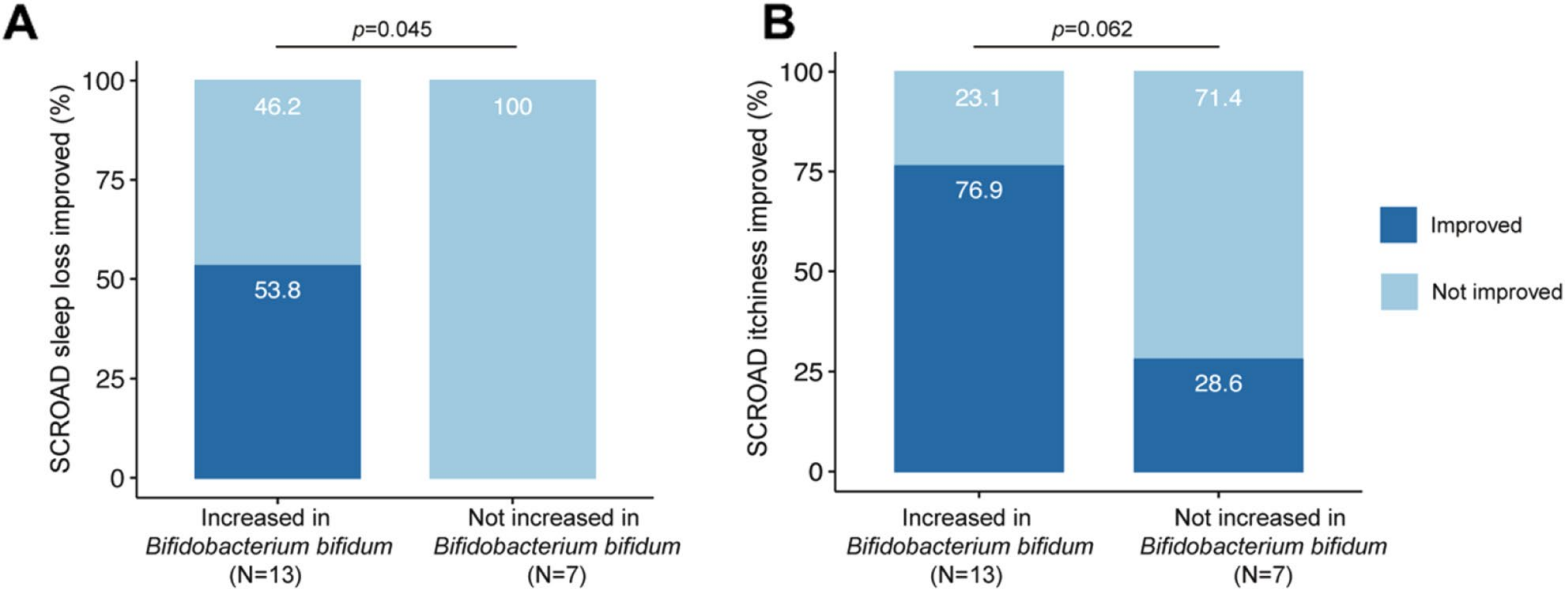
Effects of changes in stool *Bifidobacterium bifidum* after one month of SIM03 treatment and subjects’ improvement in (**A**) sleep loss and (**B**) itchiness/pruritus components of SCORAD.

**Figure 4:**
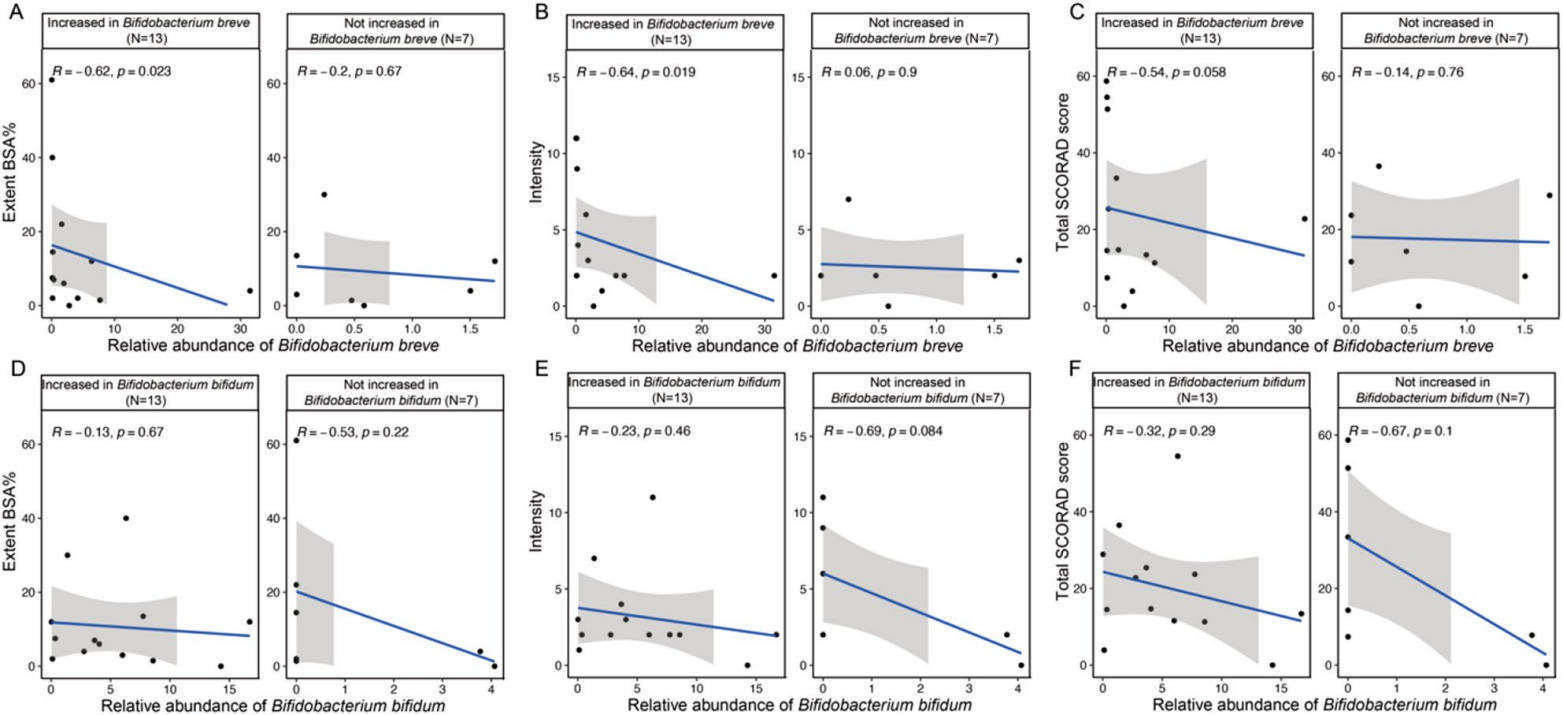
Correlations between extent of body surface area (BSA) and intensity under SCORAD as well as total SCORAD score and the relative abundances of *Bifidobacterium breve* (**A**–**C**) and *Bifidobacterium bifidum* (**D**–**F**) among subjects with and without increase in their abundances at Month 3 after SIM03 treatment.

We then compared the improvement in eczema severity between subjects with and without increase in bifidobacteria species after one-month of SIM03 treatment. At Month 1, 13 and 17 patients had increase in *B. bifidum* and *B. breve* respectively. Regarding patients who did not show increase in *B. bifidum* (n = 7), four patients had mild eczema and three had moderate-to-severe eczema at baseline. We did not analyze the results for *B. breve* due to small sample size of those without increase in its relative abundance (n = 3). More than half of the subjects with increased *B. bifidum* experienced less sleep loss while none of those with unchanged abundance of *B. bifidum* showed improved sleep (*P* = 0.045; Fig. 3A). There was also a trend towards greater improvement in pruritus score among subjects with increased *B. bifidum* than those without an increase in *B. bifidum* (*P* = 0.062; Fig. 3B). None of the SCORAD scores was significantly different between subjects with (n = 13) or without (N = 7) increase in *B. bifidum* after three months of SIM03 treatment (results not shown). Nonetheless, we found that the relative abundance of *B. breve* correlated inversely with the extent (*P* = 0.023; Fig. 4A) and intensity (*P* = 0.019; Fig. 4B) components of SCORAD as well as a trend with total SCORAD score (*P* = 0.058; Fig. 4C) among patients with increased *B. breve* at Month 3 but not in those without increase in *B. breve*. There was no significant correlation between the relative abundance of *B. bifidum* at Month 3 and total and component SCORAD scores (Fig. 4D-F).

## Discussion

This open-label clinical trial found significant improvement in eczema severity and disease-specific QoL after 3-month treatment of our novel infant microbiome formula, although such effects appeared to be independent of any change in skin biophysical paramaters. In parallel, we found a significant increase in the relative abundance in *B. breve* contained in SIM03 and enrichment in microbial pathways involved in synthesis of acetate and acetyl-CoA (precursor of butyrate) in subjects’ stool samples after one month. In particular, enhanced superpathway of menaquinol-8 biosynthesis III was found among subjects with increased *B. bifidum*. This formula appeared to be safe in our small case series, with seven unrelated adverse events and no serious adverse event being reported.

Systematic reviews and meta-analysis had demonstrated inconclusive results on the effectiveness of probiotics in treating eczema^22–24^. One possibility is the impaired delivery of ingested probiotics to lower gastrointestinal tract to alter the gut microbiota. Previous studies did not ascertain changes in stool microbiome, so it remained unknown if those probiotics were able to change the local microbial compositions in the gut. In this study, we used patented microencapsulation technology to protect the two bifidobacteria contained in SIM03 and enhance their gut delivery. Stool metagenomics indeed confirmed that this infant microbiome formula altered the abundances of *B. breve* and *B. bifidum* in our subjects’ stool samples. Other possible reasons for inconsistent benefits of probiotics included heterogeneity in study design, age of subjects, and varying species and strains as well as differences in doses and concentrations of involved probiotics. Lactobacillus and Bifidobacterium are the two most investigated probiotic species for eczema. Jiang et al. reported that mixed-strain probiotics tended to yield better effects in treating eczema^25^. Nonetheless, the strain(s) of probiotics or the optimal dose or concentration are yet to be identified. Treatment duration and timing of interventions also remain unclear. In our randomized controlled trial of 461 toddlers, we found that their stool microbiota remained unchanged following a 6-month supplementation with bioactive proteins, milk fat and 2’-fucosyllactose^26^. Thus, stool microbiome would be more resistant to dietary interventions in toddlers. Two systematic reviews suggested that probiotic treatment might be more effective among younger patients, the Asian population and in those with more severe eczema^22,23^. Due to this large heterogeneity among the studies, we need to be cautious when interpreting findings of the systematic reviews and meta-analysis.

The observation of stool microbial dysbiosis in eczema attracted great interest of designing probiotic treatment for eczema based on modulation of gut microbiota to restore the abundance of beneficial bacteria. Although there is inconclusive evidence about probiotic treatment for childhood eczema^6^, most studies reported improvement in SCORAD score and other eczema outcomes^27–30^. Tan-Lim et al. suggested that probiotic strains Mix1 (*B. animalis* subsp lactis CECT 8145, *B. longum* CECT 7347 and *Lactobacillus casei* CECT 9104) and *Lactobacillus casei* DN-114001 had the best efficacy for reducing eczema severity through network meta-analysis^31^.

*B. breve* and *B. bifidum* were shown to be effective treatment for eczema. Li et al. conducted a systematic review and meta-analysis of probiotics supplementation for eczema in adults^32^. They identified nine studies with 208 eczematous patients and 194 controls, and found *B. breve* in a mixture with *L. salivarius* (LS01) to be the optimal supplementation for treating eczema. Another study included *B. bifidum* GKB2 as one of the seven types of probiotics, and reported that this E3 probiotics formula significantly improved eczema severity^33^.

Given effective delivery and colonization of probiotics bacteria in the gut is essential to restore gut dysbiosis, we demonstrated by metagenomics sequencing the enrichment of Bifidobacterium species of SIM03 in our children. Interestingly, our subjects who showed improvement in eczema symptoms showed increased *B. bifidum* in their gut microbiota while those who did not respond to SIM03 showed no such change. Such observation was consistent with a previous study in which responders and non-responders to probiotics had distinctive microbiome signatures^33^, showing that the top five genera in gut microbiome differed between these two groups. Possibly due to our small sample size, there was no significant difference in top five genera, namely *Bacteroides*, *Bifidobacterium*, *Faecalibacterium*, *Blautia*, and *Roseburia*, between patients with or without an increase in *B. bifidum*. On the other hand, taking subjects with static *B. bifidum* as reference, four microbial pathways also increased in those with increased *B. bifidum* at Month 1 (Table S3). Individualized probiotic treatments might be necessary in eczematous patients with different stool microbiome at baseline. “Precision probiotics” are believed to serve better candidates for precision medicine, and future studies need to identify algorithms suiting person-specific data and factors affecting probiotic efficacy so that the optimal probiotic modality can be offered to stratified individuals^34^.

At the study start, about 70 percent of our subjects had bowels opened at least once daily and around half had “ideal stools” according to BSS. Following SIM03 treatment, there was insignificant change in stool frequency and consistency. Such result was compatible with similar finding from the Cochrane systematic review involving 14 clinical trials that evaluated the efficacy of probiotics for chronic constipation in 1127 children^35^. The authors could not draw any conclusion on whether there was a difference in the frequency of defecation comparing probiotics to placebo. Such analyses were limited again by heterogeneity in the definition of constipation as well as details of probiotics used and core outcome sets adopted in different clinical trials. Future clinical trials addressing this common pediatric problem should standardize these recruitment and treatment issues.

This study has several limitations. Firstly, the sample size of this study was small that precluded subgroup analysis for the effects of SIM03. The open-label nature made it impossible to blind the parents, so they might be biased when reporting changes in study outcomes during study. Furthermore, the treatment duration might not be optimal given that all children had history of long-term eczema, when a more prolonged course could be required to improve subjects’ structural skin changes. However, we were able to detect significant reductions in SCORAD scores in this 3-month study. Besides, the questions about optimal dosages of *B. breve* and *B. bifidum* and the timing of intervention remain unanswered. As subjects were 1–5 years old, it also remains unclear whether our results could be generalized to children in other age groups (e.g. infants or those older than 5 years) who consumed different diet. Despite these issues, our finding can provide important pilot data to design a sufficiently powered randomized controlled trial to elucidate the efficacy and safety of our novel SIM03 formula.

In conclusion, this clinical study illustrated the clinical benefits of SIM03 in improving eczema severity and disease-specific QoL in young eczematous children. This patented technology enhanced the delivery of probiotic bacteria to the gut, as evidenced by an early increase in fecal *B. bifidum*. SIM03 may be an effective treatment option for childhood eczema and warrants confirmation in randomized studies.

### Supplementary Information

Supplementary Information.

## Data Availability

The datasets generated and/or analyzed during the current study are not publicly available at present due to our patent application for the novel SIM03 formulation. However, the datasets will be available upon the granting of this patent or are available from the corresponding author on reasonable request.
