# Supplementary material for: A novel infant microbiome formula (SIM03) improved eczema severity and quality of life in preschool children
Source: Sci Rep. 2024 Feb 7;14:3168. doi: 10.1038/s41598-024-53848-w (PMC10850179; doi:10.1038/s41598-024-53848-w)
Supplement: Supplementary file 1 — Supplementary Information. [file 41598_2024_53848_MOESM1_ESM.docx]

**ONLINE SUPPLEMENT**

**A Novel Infant Microbiome Formula (SIM03) Improved Eczema Severity and Quality of Life in Preschool Children**

Oi Man Chan^1^*, Wenye Xu^2,3^*, Nam Sze Cheng^1^, Agnes Sze Yin Leung^1,4^, Jessica Yuet Ling Ching^2,3^, Brian Leong Yuen Fong^1^, Pui Kuan Cheong^2,3^, Lin Zhang^2,3^, Francis Ka Leung Chan^2,3^, Siew Chien Ng^2,3^, Ting Fan Leung^1,4^

^1^Departments of Paediatrics, The Chinese University of Hong Kong, Shatin, Hong Kong SAR

^2^Department of Medicine and Therapeutics, The Chinese University of Hong Kong, Shatin, Hong Kong SAR

^3^Microbiota I-Center (MagIC), Shatin, Hong Kong SAR

^4^Hong Kong Hub of Paediatric Excellence, The Chinese University of Hong Kong, Shatin, Hong Kong SAR

* Equal contributions as co-first authors

**Correspondence:**

Ting Fan Leung, MBChB, MD, FRCPCH, FAAAAI, Room 84043, Lui Che Woo Clinical Sciences Building, Prince of Wales Hospital, Shatin, New Territories, Hong Kong SAR

Tel: (852) 3505 2981. Fax: (852) 2636 0020. Email: [tfleung@cuhk.edu.hk](mailto:tfleung@cuhk.edu.hk)

**Table S1** Comparison of anthropometry and stool pattern during this 3-month study

| Parameter | Result* | | | |
| --- | --- | --- | --- | --- |
|  | Baseline | Month 1 | Month 2 | Month 3 |
| Body weight, kg | 13.4 ± 3.2 | 13.5 ± 3.2 | 13.6 ± 3.0 | 13.9 ± 3.1 |
| Standing height, cm | 93.7 ± 12.2 | 94.5 ± 13.5 | 96.3 ± 13.3 | 94.8 ± 12.7 |
| Frequency of bowel motion | | | | |
|  | Week 1-2 | Week 5-6 | Week 9-10 | Week 11-12 |
| Nil | 20.7% | 24.3% | 22.6% | 19.6% |
| Once daily | 57.1% | 55.0% | 53.9% | 55.0% |
| Twice daily | 20.0% | 19.6% | 18.9% | 21.1% |
| ≥3 times daily | 2.2% | 1.1% | 4.6% | 4.3% |
| Stool form by BSS^†^ | | | | |
|  | Week 1-2 | Week 5-6 | Week 9-10 | Week 11-12 |
| Constipation (types 1-2) | 13.2% | 11.4% | 12.8% | 16.8% |
| Ideal stool (types 3-4) | 48.6% | 53.9% | 52.9% | 55% |
| Diarrhea/urgency (types 5-7) | 13.2% | 10.0% | 11.2% | 8.6% |

*BSS* Bristol Stool Scale.

* Expressed in number (percentage) or mean ± standard deviation.

^†^ BSS was classified as type 1: separate hard lumps, like nuts (hard to pass); type 2: sausage-shaped but lumpy; type 3: like a sausage but with cracks on its surface; type 4: like a sausage or snake, smooth and soft; type 5: soft blobs with clear-cut edges (passed easily); type 6: fluffy pieces with ragged edges, a mushy stool; type 7: watery, no solid pieces, entirely liquid.

**Table S2** Microbial pathways related to butyrate biosynthesis in subjects’ stool samples between baseline and one-month after SIM03, as analyzed by DESeq2

| Pathway | baseMean | Log2FoldChange | lfcSE | stat | *P*-value | Adjusted *P*-value |
| --- | --- | --- | --- | --- | --- | --- |
| CENTFERM-PWY: pyruvate fermentation to butanoate | 1426.8 | -0.102 | 0.202 | -0.506 | 0.613 | 0.971 |
| PWY-5676: acetyl-CoA fermentation to butanoate II | 2074.7 | -0.028 | 0.146 | -0.195 | 0.846 | 0.997 |
| PWY-5677: succinate fermentation to butanoate | 170.2 | 0.057 | 0.311 | 0.182 | 0.855 | 0.997 |
| P163-PWY: L-lysine fermentation to acetate and butanoate | 0.823 | -0.296 | 2.953 | -0.100 | 0.920 | 0.997 |

**Table S3** Differential pathways between patients with or without an increase in *B. bifidum* after one-month treatment with SIM03 as analyzed by DESeq2. Patients without increased *B. bifidum* served as the reference for comparison.

| Pathway | log2 FoldChange | *P*-value | Adjusted *P*-value by FDR |
| --- | --- | --- | --- |
| PWY-7992: superpathway of menaquinol-8 biosynthesis III | 20.78 | 2.8x10^-11^ | 1.3x10^-8^ |
| METH-ACETATE-PWY: methanogenesis from acetate | 1.05 | 0.023 | 0.995 |
| PWY-7111: pyruvate fermentation to isobutanol (engineered) | 0.34 | 0.041 | 0.995 |
| PWY-6749: CMP-legionaminate biosynthesis I | 3.23 | 0.044 | 0.995 |
| UDPNACETYLGALSYN-PWY: UDP-N-acetyl-D-glucosamine biosynthesis II | 6.28 | 0.045 | 0.995 |

FDR, false discovery rate.
